# Regulation of synoviocyte activity by resveratrol in rats with adjuvant arthritis

**DOI:** 10.3892/etm.2013.1078

**Published:** 2013-04-25

**Authors:** XIAO-YU CHEN, ZHI-CHENG WANG, JUN LI, XIAO-LI LIU, YU-HUA SUN

**Affiliations:** 1 Department of Histology and Embryology, Anhui Medical University, Hefei, Anhui 230032;; 2 Laboratorial Center, Shanghai Municipal Affiliated Hospital of Shanghai University of TCM, Shanghai 200071;; 3 School of Pharmacy, Anhui Medical University, Hefei, Anhui 230032, P.R. China

**Keywords:** adjuvant arthritis, resveratrol, rat, extracellular signal regulated protein kinase1/2

## Abstract

The aim of this study was to investigate the preventive effects of resveratrol (Res) on rats with adjuvant arthritis (AA) and the mechanism(s) of action. An AA model was established by injection of Freund’s complete adjuvant (FCA). Vascular endothelial growth factor (VEGF) was visualized in joint specimens using immunohistochemistry. IL-1β and TNF-α production in synoviocytes was determined by radioimmunoassay (RIA). The mRNA expression of IL-1β and TNF-α was observed in synoviocytes using the reverse transcription (RT)-PCR method. The synoviocytes of the AA model were stimulated by Res or treated with the protein kinase C (PKC) inhibitor chelerythrine prior to stimulation. The expression of phosphorylated ERK1/2 (p-ERK1/2) was detected by western blotting. Res was able to reduce the elevated levels of IL-1β and TNF-α, and inhibit the mRNA expression of IL-1β and TNF-α in the synoviocytes of the AA model rats. VEGF expression in the Res-treated group was significantly lowered. The protein expression levels of p-ERK1/2 were significantly higher in the Res-treated group compared with those of the model group, while p-ERK1/2 was markedly lower in the group pretreated with chelerythrine. Res has a therapeutic effect on AA rats, which may be correlated with its immunoregulatory actions, and may activate p-ERK1/2 in synoviocytes via PKC.

## Introduction

Rheumatoid arthritis (RA) is a chronic autoimmune disease characterized by swelling joints, synovitis and degeneration (1). Rat adjuvant arthritis (AA), an experimental model, resembles RA in histological pathology. The progression of synovitis in AA and RA is characterized by pronounced tumor-like expansion of the synovium. Consequently, neovascularization may play a pivotal role during disease progression (2). The similarities in joint pathology between AA and RA may be employed for the screening of new drugs to treat RA (3). Non-steroidal anti-inflammatory drugs (NSAIDs), steroidal agents and immunosuppressants are usually used for the treatment of RA, but the side-effects indicate a requirement for new and more effective natural drugs. Resveratrol (3,5,4-trihydroxystilbene; Res) is a naturally occurring polyphenol present in >70 species of plants. It has been demonstrated that Res possesses anti-inflammatory, analgesic and immune-regulatory actions, and has marked preventive and therapeutic effects on AA rats (4,5). In the current study, immunology was used to observe the effect of Res on the synoviocytes of AA rats, the secretion of inflammatory cytokines and the possible mechanism(s) of action of synoviocytes.

## Materials and methods

### Animals

A total of 60 male Sprague-Dawley (SD) rats, 220–250 g, were provided by the laboratory animal center of Anhui Medical University (Hefei, China; Certificate No. 01). The rats were maintained in a colony room at an ambient temperature of 25±1°C. The lighting duration in the colony room was 12 h (from 7:00 a.m. to 19:00 p.m.). Food and water were provided *ad libitum*. The rats were acclimatized under standard laboratory conditions for 1 week prior to experimentation. The experiments were approved by the Committee of Laboratory Animals of Anhui Medical University (Hefei, China).

### Drugs and reagents

Approximately 99% pure Res was obtained from Sigma-Aldrich (St. Louis, MO, USA). A stock solution was prepared in dimethyl sulfoxide (DMSO) and further diluted in a RPMI-1640 to achieve the desired final concentration. The concentration of DMSO was 0.05% (v/v). Bacillus Calmette-Guérin (BCG) and *Tripterygium wilfordii* polyglucoside tablet (TPT; positive control compound) were purchased from Shanghai Biochemical Institute (Shanghai, China), and suspended in 0.5% sodium carboxymethylcellulose (CMC-Na) to produce the necessary concentration for the experiment. The RPMI-1640 medium was purchased from Gibco (Carlsbad, CA, USA). ^125^I-IL-1β and ^125^I-TNF-α radioimmunoassay (RIA) kits were purchased from Beijing Northern Biomedicine Company (Beijing, China), goat anti-rabbit monoclonal antibody to phosphorylated ERK1/2 (p-ERK1/2) was purchased from Cell Signaling Technology, Inc. (Danvers, MA, USA), and protein kinase C (PKC) suppressor chelerythrine from Promega Corporation (Madison, WI, USA). All other reagents were of analytical purity and were from commercial sources.

### Animal groupings and drug treatment

The SD rats were divided randomly into six groups (n=10 each), and the drug treatment groups were treated with Res intragastrically (10, 50 or 100 mg/kg/day) or the reference drug TPT (100 mg/kg/day). AA rats were created as previously described (6,7). Briefly, rats received immunization (day 0) with a single intradermal injection of 0.1 ml Freund’s complete adjuvant (FCA). FCA was prepared by mixing 10 mg heat-inactived (80°C, 1 h) BCG with 1 ml sterile paraffin oil which was then injected into the right hind foot pads of the rats. The rats in the drug treatment groups were given a continuous intragastric gavage (i.g. 10 ml/kg/day) between days 12 and 24 after immunization. For the normal and AA model rats, an equal amount of 0.5% CMC-Na solution was administered.

### AA evaluation

AA rats were evaluated as previously described (8). Briefly, the left hind paw (non-injection) volume was determined using a YLS-TA volume meter (Shandong Medicine Academy, Jinan, China) prior to immunization (basic value, day 0) and at 4-day intervals following administration of the drugs. The paw swelling (Δ ml) was defined as the increase in paw volume evaluated following inflammation.

### Synoviocyte culture

AA rats were sacrificed on the 28th day via subaxillary exsanguination under intraperitoneal anesthesia with 1% 0.2 ml sodium pentobarbital following immunization. The joint tissues were prepared by first removing the skin and separating the limb below the ankle joint. Synovium from the knee joints of the rats was excised under sterile conditions and digested with a sequential incubation of 0.2% (w/v) collagenase type II and 0.25% (w/v) trypsin. Fibroblast-like cells were isolated from affected joints by collagenase digestion, as described previously (9). Synoviocytes were resuspended with RPMI-1640 medium at a concentration of 1×10^9^ cells/l; the synoviocyte suspension (500 *μ*l) and LPS (500 *μ*l; 10 mg/l) were added to six-well plates. Following incubation at 37°C in a 5% CO_2_ atmosphere for 48 h, the supernatant containing IL-1β and TNF-α was collected and stored at −80°C.

### Immunohistochemistry

Vascular endothelial growth factor (VEGF) was visualized immunohistochemically as previously described (10). The joint specimens were initially decalcified for 2 weeks in EDTA-containing buffer and embedded in 10% formalin solution (v/v). Prior to staining, 5-*μ*m frozen sections were fixed for 30 min in ice-cold acetone. Endogenous peroxidase activity was quenched by incubating the slides for an additional 30 min in methanol (absolute) and 3% hydrogen peroxide. The slides were then incubated with polyclonal goat anti-mouse VEGF antibodies (1:500 dilution). Biotinylated rabbit anti-goat IgG (Beijing Zhongshan Golden Bridge Biotechnology Co., Beijing, China) and peroxidase-conjugated streptavidin were used as second and third reagents, respectively. The slides were incubated in the dark for 10 min at room temperature with a solution of the chromogen 3,3′-diaminobenzidine tetrahydrochloride (DAB reaction kit; Beijing Zhongshan Golden Bridge Biotechnology Co.). After rinsing with distilled water, the slides were counter-stained with Mayer’s hematoxylin.

### RIA for IL-1β and TNF-α

IL-1β and TNF-α levels in the cultured synoviocyte supernatant were assayed according to the manufacturer’s instructions (11). Briefly, a commercially available RIA kit was used. IL-1β and TNF-α were derived from the cultured supernatant. Test samples and standards of IL-1β and TNF-α were assayed in duplicate in polystyrene tubes, which were incubated at room temperature for 24 h with 100 *μ*l anti-IL-1β and anti-TNF-α antibodies. The supernatants were decanted and pellets were counted using a γ counter. Simultaneously, in certain assay samples, phosphate-buffered saline was added instead of an antibody to measure the nonspecific binding of the labeled IL-1β and TNF-α. The radioactivity of the pellets was then measured and a linear transformation curve was constructed for the calculations.

### Measurement of mRNA expression of IL-1β and TNF-α in synoviocytes

The total RNA of IL-1β and TNF-α in the synoviocytes were extracted by reverse transcription (RT)-PCR assay (12). Total RNA from synoviocytes was extracted using TRIzol, according to the manufacturer’s instructions (Invitrogen, Carlsbad, CA, USA). For each reaction, 1 *μ*g total RNA served as a template. For amplification, the size of the GAPDH, IL-1β and TNF-α products were 462, 377 and 249 bp after 30 amplification cycles, respectively. The specific primer pairs for GAPDH, IL-1β and TNF-α were GAPDH, forward: 5′-CGT GGA AGG ACT CAT GAC CA-3′ and reverse: 5′-TCC AGG GGT CTT ACT CCT TG-3′; IL-1β, forward: 5′-TTG TGG CTG TGG AGA AGC TG-3′ and reverse: 5′-GCC GTC TTT CAT ACA CAG GG-3′; and TNF-α, forward: 5′-CTG GGC AGC GTT TAT TCT-3′ and reverse: 5′-TTG CTT CTT CCC TGT TCC-3′. In all experiments, the reactions were controlled by substituting sterile nuclease-free water for the RNA template in the reaction. The PCR reactions were separated on 2% agarose gel containing 0.3 *μ*g/ml ethidium bromide and were visualized and photographed using UV transillumination. A gel electrophoresis (Bio-Rad, Hercules, CA, USA) was used for scanning and Lab Image 3.2 gel image analysis software was utilized to determine the grey ratio of the object band and internal control GAPDH standard band. The experiments were repeated at least three times for each condition.

### Western blotting examination of p-ERK1/2 in Res-treated synoviocytes

Untreated AA model synoviocytes were used as the control group. In the Res-stimulated groups, Res was added to synoviocytes from the AA model rats at concentrations of 0.5, 5 and 50 mg/l. In addition, the model synoviocytes were pretreated with chelerythrine for 2 h prior to treatment with 5 mg/l Res. The synoviocytes were maintained at 37°C with 5% CO_2_ for 24 h and then the cells were collected. Precooled cell lysate (100 *μ*l) was added per 1×10^7^ cells and the solution was mixed. The mixture was put on ice and centrifuged at 12,000 × g for 20 min. The concentration of protein, adjusted to 3 g/l, was detected by Bradford assay. Approximately 15 *μ*l solution from each sample was detected by 15% SDS-PAGE, which was blocked with 5% skimmed milk powder following overnight incubation. Anti-p-ERK1/2 and mouse monoclonal anti-β-actin (1:2,000 dilution; Sigma) were then added, the sample was maintained at 37°C for 1 h and then developed with enhanced chemiluminescence (ECL) (13). The experiments were repeated at least three times for each condition.

### Statistical analysis

The data are expressed as mean ± SD. The analysis of variance (ANOVA) was used to determine significant differences between groups. P<0.05 was considered to indicate a statistically significant result.

## Results

### Effects of Res on the histopathology of AA rat hind paws and immunolocalization of VEGF

**Figure 1.**
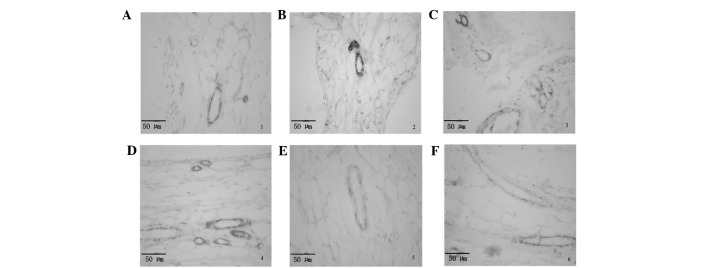
Immunohistochemical localization of VEGF in joint tissue on day 28 after the onset of arthritis (magnification, ×400). (A) Normal; (B) AA model group; (C) Res 10 mg/kg group; (D) Res 50 mg/kg group; (E) Res 100 mg/kg group; and (F) TPT 100 mg/kg group. AA, adjuvant arthritis; Res, resveratrol; TPT, *Tripterygium wilfordii* polyglucoside tablet; VEGF, vascular endothelial growth factor.

Compared with normal rats, AA rats showed hyperplastic synovium, inflammatory cell infiltration and pannus formation. These symptoms were significantly alleviated in AA rats following the administration of Res. Immunolocalization indicated that VEGF was expressed by vascular endothelial cells, but only to a small degree in normal rats (Fig. 1A). Notably, VEGF expression was markedly higher in the vascular endothelial cells of the AA model rats than in those of the normal control group, and the chondrocytes in chronically inflamed joint tissue sections showed little nonspecific staining (Fig. 1B). Compared with the AA model rats, the level of VEGF expression in the blood vessel walls of the synovium of the Res-treated and TPT-treated groups was significantly lower (Fig. 1C-F).

### Effects of Res on secondary inflammation

Inflammatory polyarthritis was induced in all immunized rats. The peak incidence occurred on the 20th day after immunization. Treatment with Res and TPT diminished the left hind paw swelling and polyarthritic symptoms on the 16th day after immunization. The suppressive effect of Res 100 mg/kg was the most marked. Res 100 mg/kg was as effective as TPT on the 24th day (Table I).

### Synoviocyte culture

The rats were sacrificed on the 28th day by subaxillary exsanguination under intraperitoneal anesthesia with 1% 0.2 ml sodium pentobarbital after immunization. The synovial tissues of the bilateral knee joints of rats were obtained under sterile conditions and the synoviocytes were cultured as previously described (8). The synoviocytes were resuspended in RPMI-1640 medium at a concentration of 1×10^9^ cells/l. After smearing the synovial membrane fibroblasts and using Giemsa stain, 300 cells were examined under the microscope. The cell purity was >95%.

### RIA assay of IL-1β and TNF-α levels in synoviocyte suspensions

**Figure 2.**
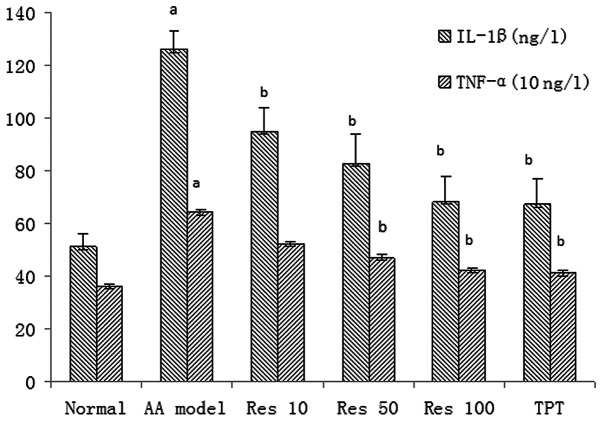
Effect of Res on IL-1β and TNF-α in secretory synoviocyte suspension (±SD, n=10). ^a^P<0.01, compared with normal group; ^b^P<0.05, compared with model group. AA, adjuvant arthritis; Res, resveratrol; TPT, *Tripterygium wilfordii* poly glucoside tablet.

Synoviocyte suspensions from the AA model rats were revealed by RIA to release higher levels of IL-1β and TNF-α than those of normal rats. Res and TPT significantly inhibited the production of IL-1β and TNF-α in the synoviocyte suspension (Fig. 2).

### Effect of different concentrations of Res on mRNA expression of IL-1β and TNF-α in synoviocytes

**Figure 3.**
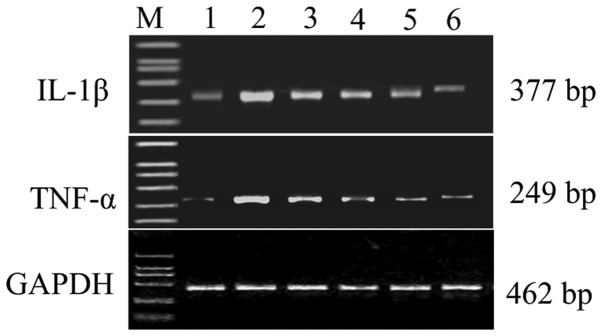
Representative gels stained for RT-PCR products of IL-1β and TNF-α mRNA expression of synoviocytes in AA model rats treated with Res. Lane M, marker; lane 1, normal; lane 2, AA model group; lane 3, Res 10 mg/kg group; lane 4, Res 50 mg/kg group; lane 5, Res 100 mg/kg group; lane 6, TPT 100 mg/kg group. RT-PCR, reverse transcription-PCR; AA, adjuvant arthritis; Res, resveratrol; TPT, *Tripterygium wilfordii* polyglucoside tablet.

In the normal group, there was weak mRNA expression of IL-1β and TNF-α, while the expression of cytokines was significantly increased in the AA group (P<0.05). As the dose of Res gradually increased from 10 mg/kg to 100 mg/kg, mRNA expression of IL-1β and TNF-α was reduced in a concentration-dependent manner. Steady-state expression of GAPDH was used to control equal loading of the PCR product onto gels (Fig. 3).

### Effect of Res on p-ERK1/2 in rat synoviocytes

**Figure 4.**
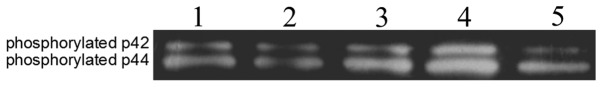
Influence of the different concentration Res on the expression of phosphorylated p42/44 ERK in AA synoviocytes. Lane 1, AA model group; lanes 2–4, stimulated by Res (0.5, 5 and 50 mg/l, respectively); lane 5, chelerythrine and 5 mg/l Res. AA, adjuvant arthritis; Res, resveratrol.

To explore the effect of Res (0.5, 5 and 50 mg/l) on p-ERK in the synoviocytes of AA model rats *in vitro*, the synoviocytes were stimulated with Res. In addition, the synoviocytes were pretreated with the PKC inhibitor chelerythrine prior to stimulation with Res. The expression levels of p-ERK1/2 in the synoviocytes were detected by western blotting. The p-ERK1/2 activity was low in the AA model synoviocytes. Following the stimulation of the synoviocytes with Res, the protein expression level of p-ERK1/2 was notably higher than that of the AA model group. Following pretreatment of the synoviocytes with chelerythrine for 2 h prior to treatment with Res, the protein expression level of p-ERK1/2 was markedly lower than that in the 5 mg/l Res-stimulated group (Fig. 4). Therefore, Res may activate p-ERK1/2 in synoviocytes via the PKC pathway.

## Discussion

RA is a chronic, systemic inflammatory disease affecting ∼1% of people worldwide. RA is a disorder characterized by persistent inflammatory synovitis, predominantly affecting the peripheral joints. The chronic nature of this disease results in progressive joint destruction, which leads to severe locomotive disability and deterioration in quality of life (1). The present study examined the therapeutic effects of Res and its mechanisms of action on AA in rats. The administration of Res during the secondary inflammation response (from days 12 to 24 after AA induction) markedly inhibited the swelling of the non-immunized hind paw. Histological examination of ankle arthritis showed that Res significantly reduced hyperplastic synovium, inflammatory cell infiltration and pannus formation. Due to the similarities in pathological features between AA and RA, these results indicate that Res may be effective in treating clinical RA (14).

Neovascularization is a complex process, involving endothelial cell division, selective degradation of vascular basement membranes and surrounding extracellular matrix, and endothelial cell migration. Several polypeptide growth factors have been identified based on their ability to stimulate the proliferation of endothelial cells in the RA joint, which include TNF-α, and acidic and basic fibroblast growth factors (12,13,15–17). Another significant mediator of neovascularization is VEGF. VEGF is an endothelial cell-specific mitogen *in vitro* and an angiogenic growth factor *in vivo*, which is known to play an important role in pathological conditions, including certain tumors and RA (13,15). However, the location and time course of VEGF expression during the development of RA remain unclear; nor has it been determined whether VEGF is directly involved in the induction of the synovitis observed in RA (16,17). Our experiments showed that Res participated in VEGF expression in the joints of AA rats and the VEGF expression levels reduced as the Res dose was increased.

Synoviocytes are the final effector cells of RA joint damage in the synovial membrane and articular cavity. Analysis shows that the mitochondrion is the energy provider for cell metabolism in synoviocytes (18–20). Our previous results showed that the secretion and metabolism of the synoviocytes of AA model rats became hyperfunctional at day 24 after immunization. The effects of Res on synoviocyte secretory function may provide an explanation for its mechanism of action in AA rats. These pharmacological effects of Res strongly suggests its potential in the treatment of autoimmune disease, particularly in RA (6).

Under normal circumstances, the expression and secretion of IL-1β and TNF-α are strictly controlled by the organism. In RA, the higher secretion of synoviocytes activated by proinflammatory factors, including IL-1β and TNF-α, is suggested to be a crucial process in the destruction of cartilaginous and bony tissues in joints affected by RA (21,22). IL-1β and TNF-α overproduction plays potential pathogenic roles in the establishment of rheumatoid synovitis, the formation of pannus tissue and the process of joint destruction. A study of experimental models revealed that IL-1β and TNF-α are key cytokines in joint swelling and cartilage destruction (23). In our study, the synoviocytes of AA rats released higher levels of IL-1β and TNF-α than those of normal rats. Res inhibited the production of IL-1β and TNF-α. RT-PCR also showed that the mRNA expression levels of IL-1β and TNF-α in AA rats were significantly increased compared with those of the normal group, and Res downregulated the mRNA expression levels of the inflammatory factors IL-1β and TNF-α.

The modulation of signal transduction by protein phosphorylation and dephosphorylation is an important regulatory mechanism. Our observations suggest that PKC-dependent p-ERK1/2 signal transduction plays an important role in AA synoviocytes, and two PKC phosphorylation sites have been identified in the present study (Fig. 4). The activity of p-ERK1/2 was low in model synoviocytes. After the synoviocytes were stimulated by Res, the protein expression levels of p-ERK1/2 were markedly higher compared with those of the model control group. When the synoviocytes were pretreated with chelerythrine, the protein expression level of p-ERK1/2 was markedly lower than those of the Res-stimulated groups.

The present study revealed the therapeutic action of Res in AA rats was associated with its ability to balance the secretion of cytokines by synoviocytes and modulate the abnormal cellular immune function, and the possible mechanism may be associated with activating p-ERK1/2 in the synoviocytes via PKC. The pharmacological effects of Res strongly suggest its potential therapeutic role for chronic RA.

## Figures and Tables

**Table I.** Effect of Res on secondary inflammatory reaction in AA rats (mean ± SD, n=10).

Group	Dose (mg/kg)	Hind paw swelling (ml)				
		Day 12	Day 16	Day 20	Day 24	Day 28
Normal	-	0.12±0.07	0.13±0.05	0.14±0.06	0.17±0.08	0.18±0.04
Model	-	0.33±0.07	0.37±0.08	0.46±0.06	0.41±0.07	0.38±0.07
Res	10	0.33±0.05	0.36±0.09	0.36±0.06^a^	0.33±0.08^b^	0.32±0.05^b^
Res	50	0.32±0.06	0.34±0.07	0.35±0.05^a^	0.31±0.06^b^	0.29±0.04^b^
Res	100	0.31±0.08	0.33±0.06^a^	0.29±0.06^b^	0.25±0.06^b^	0.23±0.05^b^
TPT	100	0.28±0.04	0.29±0.07^a^	0.28±0.08^b^	0.26±0.05^b^	0.22±0.04^b^

a P<0.05, versus model group;

b P<0.01, versus model group; AA, adjuvant arthritis; Res, resveratrol; TPT, *Tripterygium wilfordii* polyglucoside tablet.
